# Nasal Dorsum Rotation–Transposition Flap Associated with Guitar-String Sutures: One-Stage Reconstruction of Large Defects on the Nose

**DOI:** 10.3390/jcm13051404

**Published:** 2024-02-29

**Authors:** Javier Antoñanzas, Alejandra Tomás-Velázquez, Rafael Salido-Vallejo, Pedro Redondo

**Affiliations:** Department of Dermatology, University Clinic of Navarra, 31008 Pamplona, Spain; jantonanzas@unav.es (J.A.); atomasv@unav.es (A.T.-V.); rsalidov@unav.es (R.S.-V.)

**Keywords:** cutaneous surgery, nasal flap, guitar-string sutures

## Abstract

(1) **Background**: The preferred reconstructive option for closing small- or medium-sized defects of the distal half of the nose is the use of local flaps. The dorsal nasal (Rieger) flap is suitable for closing medium-sized defects at this location, especially when they are medially located, and are wider rather than tall. We describe a rotation–transposition dorsal nasal flap reconstruction for large nasal defects. The novelty of this design includes the addition of a small transposition lobe to the rotation flap, enabling the acquisition of tissue from either the lateral sidewall or the nasolabial sulcus, facilitating closure with guitar-string sutures. (2) **Methods**: We conducted a retrospective chart review of all the patients with large defects (>20 mm) of the nose who underwent nasal dorsum rotation–transposition flap repair between January 2019 and November 2022 at a single academic center. (3) **Results**: Fourteen patients (eight males, six females; ages 47–83, mean age 60 years) with defects (range: 20.00–35.00 mm) on the dorsum and nasal tip were recruited. Follow-up duration ranged from 12 months to 3 years, with all cases showing good or excellent oncologic and cosmetic results. (4) **Conclusions**: The rotation–transposition dorsal nasal flap was demonstrated to be a reproducible one-stage technique for large defects of the dorsum and nasal tip, with minimal risk of aesthetic or functional complications. Guitar-string sutures allowed the reduction of the defect size, facilitating a smaller flap design.

## 1. Introduction

The nasal dorsum is a frequent site for skin cancer, and its surgical reconstruction can be challenging, depending on the size of the defect, the location, and the individual nasal physiognomy [1,2].

While direct repair may be considered for only very small defects (<10 mm), skin grafts may be of some benefit, especially in elderly, multimorbid patients who cannot or do not wish to undergo a more complex reconstruction. However, grafts may present difficulties to take hold and undergo partial loss, especially if hematoma or infection occurs, prolonging the healing process. Moreover, despite being commonly performed following the subunit principle, grafts often yield suboptimal cosmetic outcomes [3].

Thus, the optimal choice for repairing nasal medium-sized defects (15–25 mm) is through the utilization of local flaps [4]. Flap tissue is generally recruited from either a vertical or transverse direction, depending on factors such as tissue laxity, vascularity, and resulting donor site distortion. In the reconstruction of most nasal skin defects, random pattern skin flaps are typically utilized, while axial skin flaps are selectively reserved for specific cases. Random pattern skin flaps receive their vascular supply through the musculocutaneous layer, provide by arteries that connect to the dermal plexus. Hence, meticulous design ensuring adequate perfusion pressure within the vascular network is imperative to avoid flap compromise. These considerations are particularly relevant in elderly populations, individuals with diabetes, smokers, those with limited skin laxity, or individuals with prior surgical interventions and potential impediments to flap perfusion due to scar tissue.

The reconstructed nose must harmonize with the color and texture of the original and surrounding nasal skin. Notably, the skin covering the dorsum of the nose tends to be thin and flexible, in contrast to the denser, more immobile nature of the skin over the nasal tip and ala, which are firmly adherent to the underlying framework. Consequently, the selection of tissue for reconstruction should be tailored according to the specific location of the defect. Although many flaps are described for dorsum nasal reconstruction, the dorsal nasal flap (Rieger flap), glabellar flap, bilobed flap, and V-Y advancement flap are often considered the most effective options for closing the majority of defects [5].

The dorsal nasal or Rieger flap is a local flap suitable for reconstructing defects of nasal skin in a single-stage procedure [6]. In particular, central defects located in the middle third of the nose are satisfactory addressed with a Rieger flap. The flap elevation proceeds from the upper to the lower part, beginning subcutaneously over the glabella and then submuscularly over the nasal dorsum. Advancement towards the lower part allows coverage of the primary defect, followed by closure of the upper part of the incision using a V-Y advancement. Careful consideration should be taken when employing the dorsal nasal flap for large defects or those smaller but involving the nasal tip, as this technique may result in elevation of the nose or an increase in the nasolabial angle [7]. Thus, a preferred choice for reconstruction of defects located in the distal third of the nose, particularly dome defects and central defects of the nasal tip, is by using bilobed flaps. Their design comprises two lobes of tissue, with each lobe advanced approximately 45–50 degrees, resulting in a total rotation of 90–100 degrees. The first lobe is rotated to address the primary defect, while the second digit is utilized to cover the donor site created by the rotation of the first lobe. Subsequently, the defect resulting from the rotation of the second lobe is closed primarily. An important limitation associated with the utilization of bilobed flaps is their tendency to induce asymmetry in the nasal dorsum and often also a tent effect. Conversely, diminishing the thickness of the flaps to mitigate these undesired effects may predispose to distal necrosis [7].

Lateral defects, mainly those involving vertical components, are best managed with a V-Y advancement flap from the remaining sidewall. When designing this flap, the length must be approximately 1½–2 times the longitudinal diameter of the defect. In dissecting this flap, particular care must be taken to avoid excessive detachment beyond the subcutaneous plane. Preservation of a sufficiently wide pedicle in the central and deep region of the flap is crucial to prevent necrosis of the mobilized skin.

Finally, the paramedian forehead flap is the mainstay of reconstruction for larger nasal defects [8]. It is traditionally considered an axial flap based on the supratrochlear artery. However, recent discussions have proposed that strict adherence to this arterial supply may not be essential. It has been suggested that a broad pedicle, no less than 12.00 mm in width and deep, harbors adequate anastomoses between the supratrochlear and supraorbital arteries, thereby mitigating concerns regarding pedicle compromise during rotation. Additionally, the cephalad extension of the flap can be tailored to incorporate hair-bearing scalp skin if needed to achieve sufficient length. Closure of the forehead donor site may be achieved directly for small defects; however, allowing the area to heal by secondary intention typically results in complete healing within 4 weeks. Significant limitations of the paramedian forehead flap technique are its complexity and the requirement for at least two surgical procedures. The procedure involves an initial carving and suturing of the flap into the recipient bed, followed by a subsequent intervention, approximately three weeks later, to cut the pedicle and return it back to the donor area.

An essential aspect of reconstructing defects in the nose, applicable to all the flaps mentioned above, involves preserving nasal symmetry, avoiding excessive elevation of the nasal tip, and preventing partial occlusion of the nasal fossae. Herein, we describe a series with medium to large nasal dorsum defects (20–35 mm), which were reconstructed using a one-stage dorsum rotation–transposition flap with the assistance of guitar-string sutures. This is a single-stage technique that recruits skin with excellent color and textural match, ensuring the absence of nasal asymmetry or excessive traction on the nasal tip.

## 2. Materials and Methods

We conducted a retrospective review of Mohs micrographic surgical defects on the nasal dorsum and tip repaired with a nasal dorsum rotation–transposition flap utilizing guitar-string sutures, spanning from January 2019 to November 2022 at the Department of Dermatology, Clinic University of Navarra. All patients were briefed on the nature of the reconstruction procedure and provided written informed consent. The project received approval from both the patients and the hospital’s research ethics committee.

Tumors were surgically removed using the formalin-fixed paraffin-embedded (slow Mohs) technique, resulting in a postponement of reconstructions for at least 24 h.

**Figure 1:**
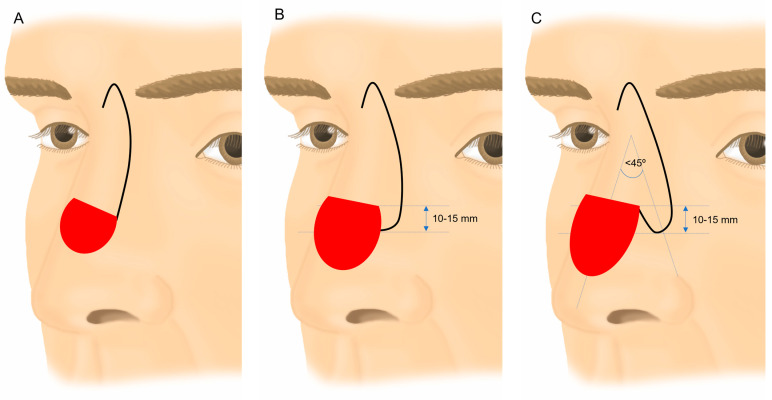
Conventional Rieger flap (**A**). Rieger flap modification with ½ lobe transposition (**B**). Rieger flap modification by associating a transposition lobe (**C**).

All interventions were performed under local anesthesia, most of them with locoregional blocks. The flaps were incised and elevated in a supraperiosteal-perichondral plane and then rotated downward to repair the dorsum and nasal tip defects. The dissecting plane included the superficial muscular aponeurotic system (SMAS) within the flap. The design of the transposition lobe or half lobe depended on the size and location of the defect. The width of the distal portion of the flap typically ranged between half and two thirds of the width of the defect, facilitating direct closure of the donor area. The length of the tab was also based on the size of the defect, usually falling within the range of 10–15 mm, measured from a tangent drawn on the upper edge of the defect. Viability was favorable, given its extension from the rotation flap with a wide, well-vascularized pedicle. When designing an entire lobe, the transposition angle with the lateral edge of the defect typically did not exceed 45° (Figure 1).

**Figure 2:**
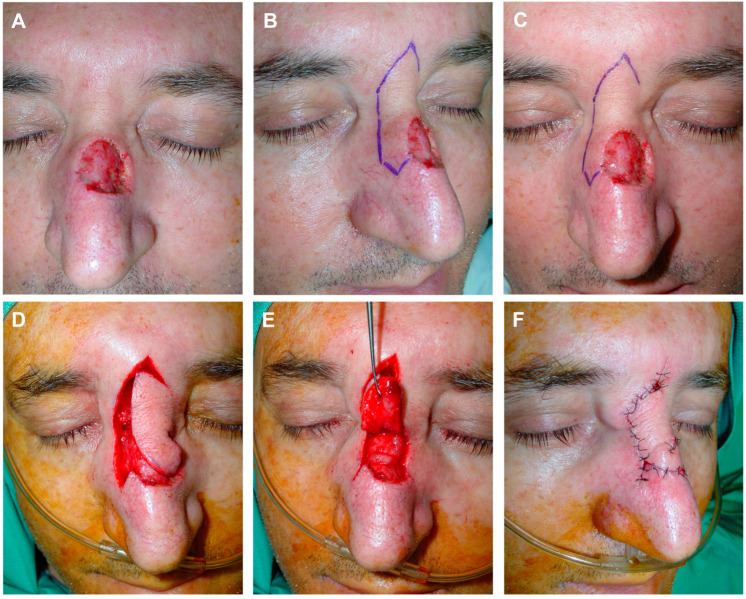
Example of guitar-strings sutures. Defect in nasal dorsum (**A**). Reconstruction design using a modified dorsal nasal flap. In the classic drawing, a finger-like portion is added, extending beyond the lateral border of the defect; this introduces a transposition movement in addition to the typical rotation of the flap (**B**,**C**). The flap is carved and advanced in a submuscular plane, revealing a disproportion between its size and the bloody area (**D**). To address this, two guitar-string sutures are made with 4/0 polyglactin from the deep subcutaneous cellular tissue of the lateral borders of the defect, reducing its size. The finger-like flap seamlessly fits over the new wound bed (**E**). Immediate result after 6/0 silk suture (**F**).

**Figure 3:**
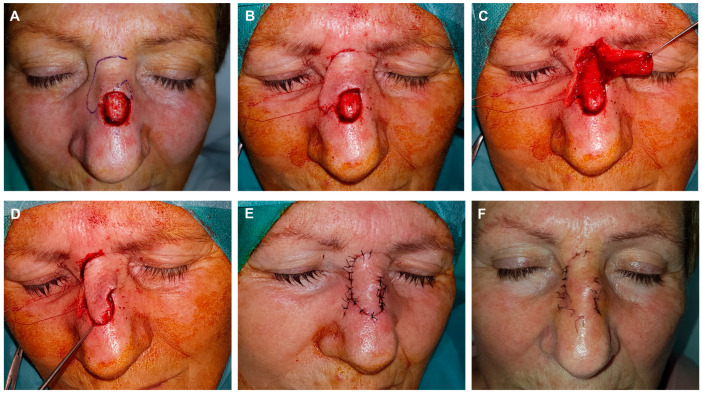
Example of guitar-strings sutures. Final defect with free margins and reconstruction with a modified dorsal flap using a horizontal glabellar incision. The design incorporates a classic dorsal rotation flap along with a small transposition flap on its distal end (**A**). The flap is dissected in the submuscular plane. A series of images illustrate the use of two guitar-string sutures to reduce the size of the defect, enabling tension-free fixation of the flap (**B**,**C**). This maneuver facilitates the tension-free insertion of the displaced tissue into the new site (**D**). Immediate result after 6/0 silk suture (**E**). Appearance after 8 days at the time of suture removal (**F**).

To diminish the size of the defects, deep subcutaneous “guitar-string” approximation stitches were employed, reducing the surface area to be covered and facilitating the suture of the lobe or distal portion of the flap without tension. The suture began at the depth of one of the wound edges, ascending towards the upper portion, crossing over to the opposite edge, where it symmetrically entered the dermis, and ended deeply. Subsequently, a traction maneuver was executed, bringing the edges in proximity without complete apposition, and the knot was fixed on one side (Figure 2 and Figure 3).

The suture used was composed of 3/0 or 4/0 resorbable synthetic material (Poliglactin 910 braided, Novosyn^®^, Braun, Barcelona, Spain), which maintains strength over the 4 weeks following the intervention. This allowed sufficient time for the wound to develop the fibrotic tissue necessary for the scar to inherently withstand tension once the suture was reabsorbed. In all patients, the size of the resulting defect after tumor removal was measured and then the area reduction after using guitar-string sutures was calculated (Table 1).

**Figure 4:**
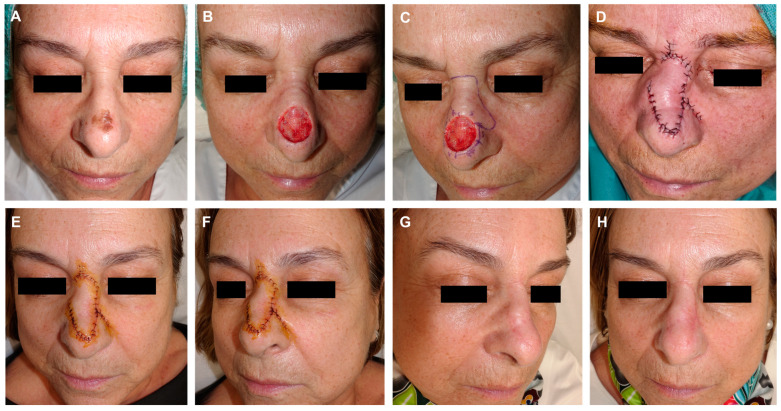
Lentigo maligna melanoma on the nasal dorsum of a 67-year-old woman (**A**). Initial defect with multiple border involvement (**B**). Lateral edges marked for removal. Design of a rotation flap from the nasal dorsum with a horizontal glabellar incision. Lateral view illustrating the dorsal rotation flap with an additional transposition movement at its tip (**C**). The flap is carved in a submuscular plane. Guitar-string sutures are used to reduce the width of the defect. Immediate result after layered sutures with 4/0 polyglactin and 6/0 silk (**D**). Appearance after 24 h (**E**,**F**). Appearance 3 months post-surgery (**G**,**H**).

**Figure 5:**
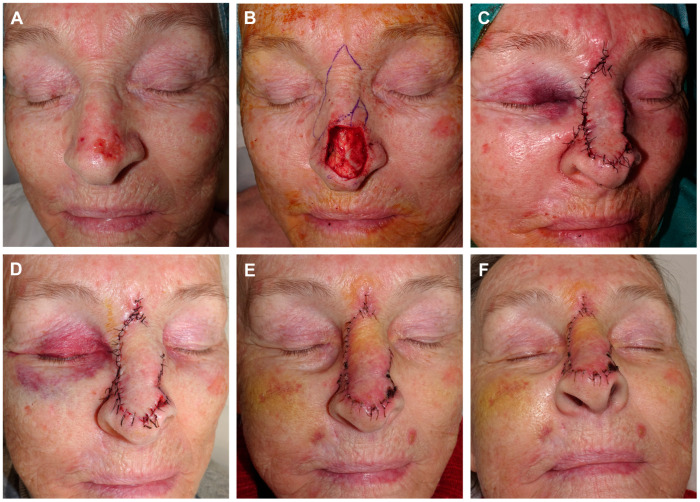
Recurrent basal cell carcinoma on the nasal dorsum and tip (**A**). Final defect after a three-dimensional histology study. Modified dorsal flap with a small transposition flap from the nasolabial junction. A Burow’s triangle on the superior border to facilitate tissue movement (**B**). Immediate result after layered closure (**C**). Result after 24 h (**D**). Appearance 6 days later at suture removal (**E**,**F**). The combination of a dorsal rotation flap with a finger-like transposition flap allows for complete coverage of large defects on the distal nasal tip. The initial elevation of the nasal tip corrects spontaneously.

During the movement of the tissue (rotation + transposition), a Burow’s triangle was formed at the pivot point. It was removed superficially, including the epidermis and dermis, but not the subcutaneous cellular tissue, to prevent unwarranted damage to deeper vessels that could compromise the viability of the flap (Figure 4 and Figure 5).

## 3. Results

**Figure 6:**
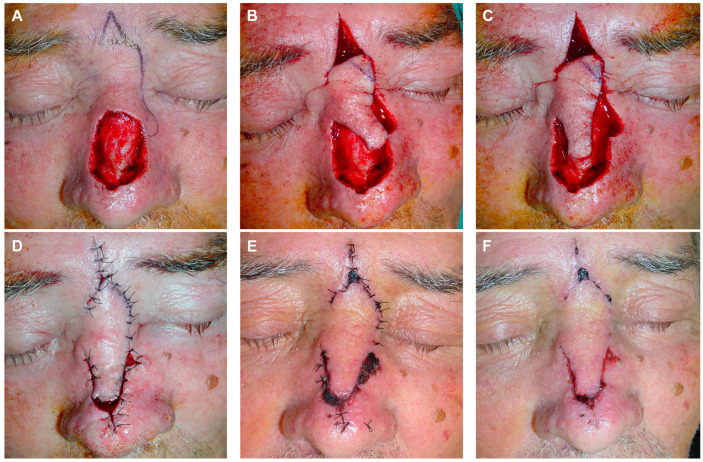
Large defect on the nasal dorsum. Design of a modified dorsal flap ending in a small transposed digit on the lateral nasal skin (**A**). Flap carved in the submuscular plane, displaced to reconstruct the defect (**B**). Significant disproportion between the width of the flap and the width of the defect (**C**). Immediate closure following the use of two guitar-string sutures, reducing the size of the defect and enabling tension-free flap assembly. Small bloody areas that will heal through secondary intention (**D**). Appearance after 72 h (**E**). Final result after suture removal at 7 days (**F**).

Fourteen patients (eight males, six females; ages 47–83, mean age 60 years) with defects ranging from 20.00 mm to 35.0 mm on the dorsum and nasal tip were included. The causes of the defects were basal cell carcinoma in ten patients, squamous cell carcinoma in two patients and lentigo maligna in the remaining two cases. As they were located at the nose, a high-risk location for dissemination, all the tumors were extirpated using Mohs micrographic surgery. In eight patients, free margins were achieved after a single passe, however in the other six patients, additional stages were needed to completely remove the tumors. As a consequence of the large defects size, at least 20 mm wide, all patients underwent reconstruction using the described rotation–transposition flap of the nasal dorsum. In addition, we used guitar-string sutures in the hole series, that resulted in a reduction of the bloody area by 15–45% (Figure 6).

In the immediate postoperative period and initial days, most patients exhibited marked local inflammation, slight nasal asymmetry, and tip elevation, which gradually subsided in the subsequent weeks. There were no cases of wound infection, suture dehiscence, noticeable scarring, or pigmented changes. In two patients, there was a mild distal suffer of the flap at the nasal tip of the nose, that resulted in crusting that was solved satisfactorily after local cures with Vaseline ointment. The follow-up duration ranged from 12 months to 3 years. The evolution in all 14 cases was favorable, demonstrating proper healing, a satisfactory aesthetic outcome, and no signs of tumor recurrence. Scar revision was not required for any patient.

## 4. Discussion

The classic nasal dorsum flap involves advancement of dorsal nasal skin from the proximal two-thirds of the nose and glabella to cover a defect of the distal nose, especially when they are located medially. This technique, originally described by Gillies, was popularized by Rieger, who in 1967 described it as a useful random flap for repairing defects not exceeding 20.00 mm in diameter located in the distal half of the nose [6].

In 1985, Marchac and Toth [8,9,10] modified the Rieger flap by introducing an axial pedicle based on vessels near the medial canthus (axial frontonasal flap). This modification allows for significant sliding and rotation of the nasal skin located above the defect with immediate precise adjustment. The donor site can be closed in a V-Y manner, with the donor scar lying in a glabellar frown line. Finally, Rohrich and colleagues [11] suggested that the standard glabellar incision was not necessary for extending or rotationally advancing the flap, especially as the resulting glabellar scar or web may detract from the final aesthetic results, and they proposed the use of a transverse incision in the radix crease as an alternative [12].

**Figure 7:**
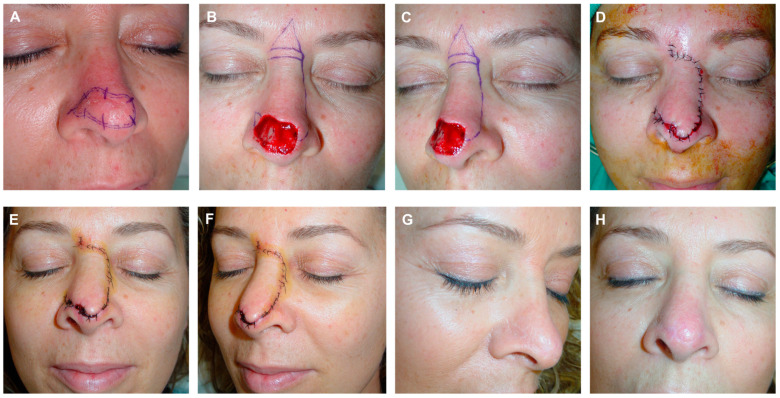
A 57-Year-old woman with poorly delimited basal cell carcinoma on the dorsum and nasal tip (**A**). Excision margins for three-dimensional histology study in paraffin. Final defect. Design of a modified dorsal flap including a slight transposition movement, with a horizontal incision at the glabella. Burow’s triangle facilitating tissue movement (**B**,**C**). Flap carved in a submuscular plane. Immediate result after layered suture. Small bloody areas expected to heal by secondary intention (**D**). Appearance 6 days post-surgery (**E**,**F**). Result 6 months post-surgery (**G**,**H**).

**Figure 8:**
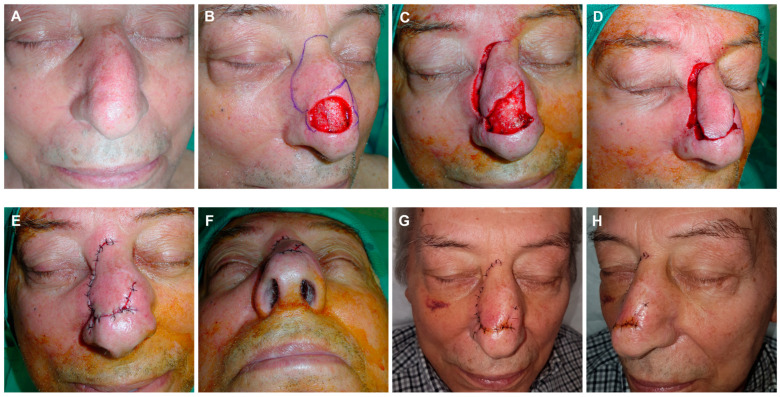
Recurrent lentigo maligna on the nasal dorsum of a 72-year-old man (**A**). Excision margins for three-dimensional histology study. Initial defect with two positive borders. Design of a dorsal flap with a horizontal glabellar incision. Flap modification with a small finger-like transposition flap on the lateral sidewall. Superior Burow’s triangle to facilitate tissue movement (**B**). Flap carved in a submuscular plane. Final defect after Burow’s triangle and lateral borders removed (**C**). Guitar-string suture to reduce the width of the defect (**D**). Immediate result after layered closure (**E**,**F**). Appearance 6 days after surgery (**G**,**H**).

**Figure 9:**
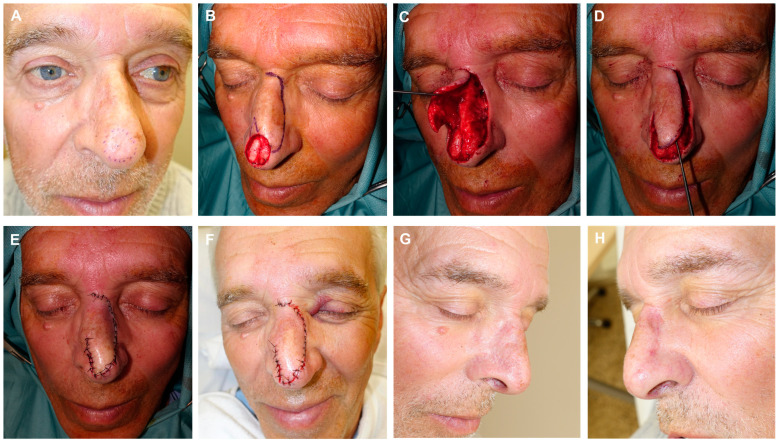
Basal cell carcinoma on the nasal tip (**A**). Final defect after two Mohs stages. Design of a dorsal nasal rotation flap with minimal transposition and a horizontal incision at the glabella (**B**). Flap carved at the submuscular plane and displaced with a skin hook (**C**,**D**). Immediate result after suturing in layers (**E**). Appearance at 24 h (**F**). Aspect at 6 days at suture removal (**G**,**H**).

In dermatological surgery, three classical random flap types are described based on skin displacement or movement: advancement, rotation, and transposition flaps [13]. However, these movements are occasionally not purely isolated, leading to the consideration of combined flaps. In this article we introduce a modified Rieger flap variant, that combines a transposition digit with the conventional rotation flap, to address large defects on the nasal dorsum and those located at the tip. This technique was previously partially described by our group for application in nasal procedures [14]. Similar to the classic dorsal nasal flap, the current modification is designed for the direct closure of the donor area, offering flexibility for either a V-Y suture in the glabella or a horizontal incision in the nasal bridge. The flap must be meticulously carved in the deep perichondral subperiosteal plane, encompassing the nose muscles to ensure vascular supply and guarantee viability. The novelty of this variant lies in an adjusted rotation flap design with an increased length of the flap leading to the edge to compensate for tethering. In this article we better define the use of half a digit (Figure 7, Figure 8 and Figure 9) or a whole digit of transposition (Figure 4, Figure 5 and Figure 6), depending on the width and height of the defect.

Based on our experience, this flap proves useful for addressing distal and medial defects of the dorsum and nasal tip, typically rectangular and taller rather than wide, where the classic Rieger flap may be insufficient. Another notable improvement is the incorporation of guitar-string sutures made from resorbable material, resulting in a significant reduction in the original defect size and subsequently narrowing the flap design.

The term “guitar-string suture” was described in 2014 [15], owing to its resemblance to the tension supported by the strings of the musical instrument. It refers to subcutaneous points that approximate the farthest edges of the surgical defect without directly facing them, thereby reducing the extension of the bloody surface and adapting it to the size of the designed digit. Although the guitar-string suture may seem conceptually similar to a suspension suture, they are different. The basis of a suspension suture involves attaching the displaced skin to a deeper fixed point, typically the periosteum, acting as an anchor to prevent undesired facial asymmetries resulting from the displacement of structures such as the eyelids, nose, or lips [16]. Conversely, the guitar-string suture approximates two equally movable areas of skin, identified as the edges of the defect furthest from each other. Thus, while the objective of a suspension suture is to anchor the skin to prevent distortions of facial symmetry, the guitar-string suture aims to reduce the size of the defect. The technique of suturing with a guitar-string stich does not significantly differ in execution from a conventional subcutaneous suture. The primary distinction lies in the context of wound closure. While a conventional subcutaneous suture aims to completely close the surgical wound by bringing the edges of the defect into direct contact, a guitar-string suture is employed when dealing with larger defects that cannot be closed directly. In such cases, the knot is tied in the center of the defect, reducing its dimensions but not achieving complete closure. Consequently, this approach allows for coverage with a smaller flap size, potentially necessitating less mobilization of surrounding skin, which is of special interest in visible areas and without excessive skin laxity as occurs in the series herein we present.

Regarding potential disadvantages, there is a theoretical concern that suturing guitar strings could introduce a foreign body into the skin, potentially increasing the risk of infection. However, in our experience, we did not observe an elevated risk of postoperative infection in any of the cases. It is important to note that the sutures are placed in a deep plane without excising the edges of the defect, resting on the bloody bed to prevent the creation of a dead space that could promote a tent effect [17]. In our series, notable complications related to the flap were not observed. However, one potential limitation could arise from a suboptimal design of the distal part of the flap to be transposed. If this portion is excessively large or located in no lax skin, it may hinder direct closure of the donor area, needing advancement of the ipsilateral cheek to address it with the consequent nasolabial fold asymmetry. Additionally, failure to carve the flap in the deep plane or to maintain a sufficiently wide pedicle could lead to perfusion issues, particularly at the distal area of the flap causing necrosis. However, when the technique is well designed and executed as specified in the methods section, none of these complications may occur and the results, from our opinion, are quantitatively and qualitatively excellent.

## 5. Conclusions

In conclusion, we presented a modified Rieger flap variant, to address large defects on the nasal dorsum and tip in a single procedure with no asymmetries or nasal elevation. The innovation in this approach lies in the combination of a small transposition digit to the rotation flap, along with the use of guitar-string sutures. This technique resulted in a reduced final surface area of the bloody region, allowing the fitting of a smaller flap lobe without tension. Direct closure of the donor zone was achieved through the rotation plus transposition movement, preventing ala traction and asymmetry of the nasal pyramid. It has an appropriate design, the procedure is uncomplicated, easy to perform, and enables subtle refinement of the nasal dorsum, achieving a aesthetically pleasing and stylized nose.

## Figures and Tables

**Table 1:** Population data and tumor characteristics of the series.

	Gender	Age	Nasal Location	Cause	Size	Reconstruction	Area Reduction by Guitar-String Sutures	Complications	Follow-Up (Months)	Recurrence
1	F	47	Dorsum	BCC	20	NSDTF	15	NO	12	NO
2	F	52	Dorsum	BCC	24	NSDTF	22	NO	15	NO
3	M	66	Tip	SCC	32.5	NSDTF	25	NO	22	NO
4	M	74	Dorsum	LMM	30	NSDTF	30	NO	36	NO
5	F	58	Dorsum	LMM	28	NSDTF	45	NO	30	NO
6	F	77	Dorsum	BSC	27	NSDTF	20	NO	25	NO
7	M	62	Tip	SCC	25	NSDTF	25	NO	24	NO
8	M	47	Dorsum	BCC	28	NSDTF	30	NO	14	NO
9	M	71	Dorsum	BCC	30	NSDTF	35	Minimal distal flap suffering	16	NO
10	M	57	Tip	BCC	31	NSDTF	20	NO	22	NO
11	F	64	Tip	BCC	24	NSDTF	40	NO	36	NO
12	F	72	Dorsum	BCC	28	NSDTF	30	Minimal distal flap suffering	30	NO
13	M	58	Dorsum	BCC	27	NSDTF	30	NO	28	NO
14	M	66	Tip	BCC	29	NSDTF	15	NO	14	NO

BCC: basal cell carcinoma; LMM: lentigo maligna melanoma; SCC: squamous cell carcinoma; NSDTF: Nasal dorsum transposition flap.

## Data Availability

Supporting data can be provided by contacting with the corresponding author.
